# Natural history of incidentally diagnosed prostate cancer after holmium laser enucleation of the prostate

**DOI:** 10.1371/journal.pone.0278931

**Published:** 2023-02-02

**Authors:** Jang Hee Han, Dae Hyuk Chung, Min Chul Cho, Ja Hyeon Ku, Chang Wook Jeong, Cheol Kwak, Jae-Seung Paick, Seung-June Oh

**Affiliations:** 1 Department of Urology, Seoul National University Hospital, Seoul, South Korea; 2 Department of Urology, SMG-SNU Boramae Medical Center, Seoul, South Korea; 3 Department of Urology, Seoul National University College of Medicine, Seoul, South Korea; 4 Department of Urology, Mediplex Sejong Hospital, Seoul, South Korea; Chung Shan Medical University, TAIWAN

## Abstract

**Objectives:**

There is no consensus on the management plan for incidental prostate cancer (IPCa) after holmium laser enucleation of the prostate (HoLEP). This study aims to investigate the natural course of this disease and suggest appropriate treatment in real clinical practice.

**Methods:**

The medical records of a prospective cohort of patients with LUTS/BPH who underwent HoLEP between July 2008 and December 2020 at Seoul National University Hospital were retrospectively reviewed. Patients who underwent HoLEP for palliative purpose of prostate cancer control were excluded. The natural history of IPCa was assessed by the clinician in a descriptive manner for each treatment option.

**Results:**

Among 2630 patients, 141 (5.4%) were diagnosed with IPCa after HoLEP. Pathologic T stage and magnetic resonance imaging results were highly associated with the physician’s primary treatment decision-making for IPCa. Active surveillance (AS) was performed in 80% of patients, of whom 90% underwent follow-up without intervention, while the remaining 10% underwent deferred active treatment with a median follow-up of 46.3 months due to International Society of Urological Pathology grade group upgrading or increasing core involvement percentage. Meanwhile, 20% of patients underwent immediate active treatment. With a median follow-up period of 88.3 months after treatment, only one of 25 patients had biochemical recurrence.

**Conclusions:**

The incidence of IPCa after HoLEP was 5.4%, and among these, approximately 20% proceeded with immediate definitive therapy and an additional 6% ultimately received definitive therapy within a median of 4 years of AS but showed excellent oncological outcomes.

## 1. Introduction

Benign prostatic hyperplasia (BPH) affects approximately 70% of men by the seventh decade of life in the United States; among them, approximately 1% of patients ultimately undergo active intervention [1], mostly transurethral endoscopic surgery, such as transurethral resection of the prostate (TURP), and holmium laser enucleation of the prostate (HoLEP). Theoretically, HoLEP shares the surgical principle of enucleation, which allows complete removal of adenoma tissue in the transition zone. HoLEP is currently accepted as the gold standard for all prostate sizes [2, 3].

Prior to prostatectomy in patients with BPH with prostate-specific antigen (PSA) level elevation, one of the critical steps is the preoperative exclusion of prostate cancer (PCa). With the widespread use of PSA screening and the development of imaging techniques, such as multiparametric prostate magnetic resonance imaging (mpMRI) [4], the incidence of incidental PCa (IPCa) detected in HoLEP seems to have decreased [5]. However, it still occurs in approximately 8% of cases in the final pathology examination [5]. Moreover, in some respects, clinicians insist that the incidence of IPCa has increased compared to the previous TURP era due to removal of the entire transition zone along the surgical capsule [5, 6].

Despite the considerable incidence rate, there are no clinical guidelines regarding the management of this group of patients for several reasons. First, most studies were conducted with a small number of patients, even when mixed with both TURP and HoLEP cases [5]. Second, most studies focused on the discovery of predictive factors for IPCa rather than its long-term oncological outcome, lacking data on the real-world IPCa management practice.

Therefore, this study aimed to primarily focus on the natural history of IPCa after HoLEP. It attempted to assess how clinicians make their decisions and how each decision led to long-term oncological outcomes in a descriptive manner.

## 2. Materials and methods

### 2.1 Ethics approval and informed consent

This study was approved by the Institutional Review Board (IRB) of Seoul National University Hospital (SNUH) (IRB no. 2201-084-1290). The requirement for informed consent was waived owing to the retrospective nature of the study. The study was performed in accordance with applicable laws and regulations, good clinical practice, and ethical principles provided in the Declaration of Helsinki.

### 2.2 Patient population

The medical records of consecutive patients with LUTS/BPH who underwent HoLEP from July 2008 to December 2020 at SNUH were obtained from our prospective BPH cohort. Patients who underwent HoLEP for the palliative purposes of PCa control were excluded.

### 2.3 Collected parameters

We collected and reviewed the following items: patient’s demographic and clinicopathological data, including age at surgery, accompanying comorbidities, preoperative transrectal ultrasonography, MRI findings, PSA level, digital rectal examination findings, preoperative prostate biopsy results, perioperative findings (operative time, resection time, resection volume, used energy, intraoperative complications, and hospital stay), pathologic findings, follow-up data with prostate biopsy, prostate MRI, PSA level, treatment information of IPCa, and oncological outcomes.

### 2.4 Statistical analysis

All statistical analyses were performed using SPSS version 25 software (SPSS, version 25.0.0.2, IBM Corp., Armonk, NY, USA). A P-value < 0.05 was considered statistically significant, and all statistical tests were two-sided.

## 3. Results

### 3.1 Patient characteristics

A total of 2630 patients who underwent HoLEP were included in this study. Their median PSA level was 2.7 ng/mL, and the mean prostate volume (PV) was 67.5 g. Among them, 141 (5.4%) were pathologically proven to have IPCa (Table 1). In the IPCa patient group, approximately 30% of patients underwent preoperative prostate biopsy, while 13% of patients underwent concurrent prostate biopsy with HoLEP. The median PSA level was 3.3 ng/mL. Most patients were classified in the International Society of Urological Pathology (ISUP) grade group (GG) 1 (85.1%), followed by GG 2 (12.8%). Moreover, there were three patients (1.4%) who showed higher than GG 3. According to the D’Amico risk stratification, approximately 79% were classified in the very low to low risk group, while the remaining 21% belonged to the intermediate to high risk group. The median follow-up period was 48.9 months (Table 2).

**Table 1:** Baseline characteristics.

Total (n)	2630
Age (years)	69.8±7.3
BMI (kg/cm^2^)	24.2±3.1
PSA (ng/ml) †	2.7 [1.5, 5.1]
PSA density (ng/ml^2^) †	0.05 [0.03, 0.07]
Total prostate volume (TPV)	67.5±33.9
Transition zone volume (TZV)	38.7±25.9
Enucleated weight (g)	24.9±25.5
Enucleated volume / TPV (%)	33.9±27.9
Enucleated volume / TZV (%)	62.5±60.5
Pathology (n,%)	
Benign prostatic hyperplasia	2482(94.3)
Prostate cancer	141 (5.4)
PIN / ASAP	2 (0.1)
Transitional cell carcinoma	2 (0.1)
Other cancers	3 (0.1)

†Data are presented as median (interquartile range)

PSA, prostate-specific antigen; PIN, prostate intraepithelial neoplasia; ASAP, atypical small acinar proliferation

**Table 2:** Characteristics of incidental prostate cancer.

Total (n)	141
Age (years)	72.1±6.6
PSA (ng/ml) †	3.3 [1.8, 5.8]
PSA density (ng/ml^2^) †	0.05 [0.03, 0.09]
Total prostate volume	65.2±35.1
Prostate biopsy prior to HoLEP	42 (29.8)
Concurrent Prostate biopsy with HoLEP	18 (12.7)
T stage (n, %)	
T1a	125 (88.7)
T1b	14 (9.9)
ISUP Grade Group	
Grade Group 1	120 (85.1)
Grade Group 2	18 (12.8)
Grade Group ≥3	3 (1.4)
Risk stratification	
Very low to Low risk	111 (78.7)
Intermediate risk	29 (20.6)
High risk	1 (0.7)
Median follow up period (months)	48.9 [23.5, 77.9]

†Data are presented as median (interquartile range)

PSA, prostate specific antigen; HoLEP, holmium laser enucleation of the prostate

### 3.2 Comparison of clinical features between T1a and T1b

Of patients with IPCa, 125 (88.7%) had T1a, and 14 (9.9%) had T1b (tumor volume percentage was not annotated in two cases). Age, PSA level, and ISUP GG were not significantly different between the two groups. In T1b, total PV tended to be smaller (p = 0.069), and PSA density tended to be higher (0.07 vs 0.05, p = 0.054) with borderline significance compared to T1a. Patients with T1b were more likely to undergo active treatment than active surveillance (AS) (S1 Table).

### 3.3 Comparison of clinical characteristics between two different decision groups: AS and immediate active treatment group

Of 141 patients, 18 were lost to follow-up, leaving 123 patients for analysis. Among these, 98 patients (79.7%) underwent AS, whereas 25 patients (20.3%) underwent immediate active treatment. Age, PSA level, and ISUP GG were comparable between the two groups. Clinicians tend to choose active treatment when the tumor volume exceeds 5% of the total specimen (T1b) or when a tumorous condition is identified on postoperative mpMRI. Relatively low prostate volume or high PSA density was also associated with the physician’s intent to treat actively with borderline significance (Table 3).

**Table 3:** Clinician’s decision toward active surveillance or immediate treatment.

Number of patients (n)	Active Surveillance (N = 97)	Active Treatment (N = 25)	p-value
Age (years)	71.7±6.4	71.4±6.6	0.808
PSA (ng/ml) †	3.3 [1.9, 5.3]	3.3 [1.9, 6.8]	0.800
PSA density (ng/ml^2^) †	0.05 [0.03, 0.07]	0.07 [0.10, 0.14]	0.097
Total prostate volume	68.7±38.1	54.7±25.2	0.085
T stage (n, %)			<0.01
T1a	90 (94.7)	18 (72.0)	
T1b	5 (5.3)	7 (28.0)	
ISUP Grade Group			0.111
Grade Group 1	84 (86.6)	19 (76.0)	
Grade Group 2	12 (12.4)	4 (16.0)	
Grade Group ≥3	1 (1.0)	2 (8.0)	
Abnormal MRI (n, %)	36 (43.4)	20 (80.0)	<0.01

†Data are presented as median (interquartile range)

PSA, prostate-specific antigen

### 3.4 Characteristics and oncological outcome of the active treatment group

Among 25 patients, radical prostatectomy was the most commonly performed treatment (n = 18, 72%), followed by definitive radiation therapy (n = 5, 25%). Pathological findings showed that 89% of tumors had multifocality, and ISUP upgrading was observed in 27.8%. Meanwhile, no residual tumor was found in one patient. With a median follow-up period of 88.3 months after treatment, biochemical recurrence (BCR) occurred in one patient (S2 Table).

### 3.5 Characteristics of patients who discontinued AS

Among patients who chose AS as primary therapy, 88 continued AS with a median follow-up period of 41.6 months, while nine discontinued AS after a median follow-up period of 46.3 months. The AS discontinuation group showed a higher ISUP GG and higher proportion of abnormal mpMRI findings. Moreover, the total PV tended to be smaller in the AS discontinuation group (p = 0.056), while PSA or PSA ratio (post-HoLEP/pre-HoLEP) did not differ between the two groups (Table 4). The most common reason for AS discontinuation was ISUP GG upgrading following prostate biopsy (n = 5, 55.6%), followed by increasing core involvement percentage (n = 3, 33.3%) (Table 5).

**Table 4:** Characteristics of patients who discontinued active surveillance.

Number of patients (n)	AS continue (N = 88)	AS discontinue (N = 9)	p-value
Age (years)	71.9 [68.1, 76.1]	70.9 [67.9, 75.6]	0.486
PSA (ng/ml)	3.4 [1.9, 5.6]	2.8 [2.3, 4.4]	0.816
PSA ratio (post/pre)	0.27 [0.18, 0.53]	0.34 [0.26, 0.70]	0.111
PSA density (ng/ml^2^)	0.05 [0.03, 0.07]	0.06 [0.03, 0.10]	0.550
Total prostate volume	62.4 [48.4, 82.0]	50.9 [40.7,54.9]	0.056
T stage (n, %)			0.399
T1a	82 (95.3)	8 (88.9)	
T1b	4 (4.7)	1 (11.1)	
ISUP Grade Group (n,%)			0.039
Grade Group 1	77 (87.5)	7 (77.8)	
Grade Group 2	11 (12.5)	1 (11.1)	
Grade Group ≥3	0 (0.0)	1 (11.1)	
Follow-up period	41.6 [26.5, 67.6]	50.3 [37.7, 93.2]	0.087
Follow-up period		46.3 [24.7, 93.6]	
Until AS discontinue			

PSA, prostate-specific antigen; AS, active surveillance

**Table 5:** Reason for AS discontinuation (N = 9).

Total (n)	9
ISUP Grade group upgrading	
GG1 → GG2	2 (22.2)
GG1 → GG3	1 (11.1)
GG1 → GG5	1 (11.1)
GG2 → GG4	1 (11.1)
Core involvement percentage ≥ 50%	3 (33.3)
Abnormal MRI and PSA velocity increased	2 (22.2)
Patient wants	1 (11.1)

†Data are presented as median (interquartile range)

PSA, prostate-specific antigen

## 4. Discussion

Previous reports have defined IPCa as a low-grade, indolent disease that does not require subsequent intervention [1, 6–8]. Thus, most patients were considered reliable candidates for AS [9, 10]. However, due to the small number of patients included in each study and relative short follow-up time with absence of subsequent treatment description (treatment plan was unknown for approximately 40% of previous studies [5]), no clinical consensus could be made. In the real-world setting, there are still controversies regarding the risk of progression of IPCa, which is the most appropriate management for these patients [5].

Moreover, several additional points emphasize the necessity of this study. Since the AS criteria have been established [11], there has been a trend of continuous increase in AS [12–14], based on the hypothesis that low-risk PCa is indolent and cancer-specific mortality is lower than other-cause mortality. However, these criteria are based on prostate biopsy results rather than on specimens acquired from minimally invasive endoscopic surgery. We still cannot confirm whether we can apply the same AS criteria, based on a previous study that showed that transition zone PCa has a different biology than peripheral zone cancer [13, 15, 16]. Thus, we tried to comprehensively demonstrate the natural course of IPCa using prospectively registered large population-based cohorts with long-term follow-up.

In this study, we focused on several issues that have been less investigated in previous studies. Our study cohort had relatively low PSA and PSA density levels compared with those in previous studies, indicating that PCa was more sensitively screened before surgery. For the similar reason, PSA reduction ratio at 6 months (PSA 6months/Pre-operative PSA) after surgery was not significantly different between IPCa and BPH patients (IPCa 0.33 [0.22, 0.58] vs BPH 0.29 [0.16, 0.57], p = 0.091).

The first issue we demonstrate was the descriptive feature that affects the clinician’s decision to perform AS or active treatment in a real-world setting. We revealed that approximately 80% of patients with IPCa underwent AS, while 20% of patients underwent active treatment. We clearly demonstrated that clinical stage T1b disease and abnormal mpMRI results were associated with immediate active treatment (Table 3). To be more specific, among 25 patients of active treatment, 20 patients (80%) showed suspicious prostate cancer lesions (16 peripheral zone, 4 transitional zone) on prostate MRI at a median of 1.2 months after HoLEP surgery. Among them, 7 patients also harbored a higher pathological tumor burden (>5%). For the other 5 patients who did not show abnormal findings on MRI, most of them showed clinically significant PCa in either HoLEP specimen or prostate biopsy performed post-operatively, which may have led to immediate active treatment. This decision was supported by previous studies showing that T1b is an independent predictor of BCR and disease-specific mortality [17, 18] and that patients with undistinguishable lesions on mpMRI should be considered for AS because they have the least risk of residual cancer development after TURP [19]. Furthermore, as in a previous study [20], high PSA density was also associated with active treatment decisions, while PSA level did not show any difference, probably due to active exclusion of suspicious patients with PCa preoperatively. Interestingly, PCa volume percentage (pathologic T stage) had a greater impact than ISUP GG on decision making.

The second objective was to demonstrate the characteristics of T1a and T1b. PCa is now being more widely screened preoperatively in the real-world setting with the help of PSA screening and mpMRI, thus increasing the prostate biopsy performance. For this reason, contradictory to the previous study by Capitanio et al., which showed small PV with high PSA in patients with T1b compared to patients with T1a [7], our cohort showed higher PSA density in T1b with borderline significance (p = 0.054), while having comparable results for PSA level and PV. Because of the small number of patients who underwent AS in T1b, we could not compare the prognosis between T1a and T1b patients who underwent AS.

The third issue was to comprehensively describe the natural history of patients who underwent AS. Of 97 patients who primarily underwent AS, approximately 90% maintained AS with a median period of 41.6 months, while 10% discontinued AS after a median period of 46.3 months. This AS continuation and discontinuation ratio was comparable to that in the HAROW study, showing 12.1% of AS discontinuation [21], and these rates are lower than those reported from most AS trials with a general low-risk PCa population [10]. This indicates that the patient may be informed preoperatively that AS may be safely recommended, and after 4 years, with regular check-up, residual cancer may show progression, such as GG upgrading or increased core involvement. However, even in these cases, cancer may be safely controlled. Furthermore, it is interesting to note that a small prostate is associated with AS discontinuation, and it is noteworthy that it is also known as a predictor of IPCa preoperatively in many studies [5].

The fourth issue was determination of whether the immediate active treatment group was truly worthy of active treatment rather than AS. For a median period of 7.5 years, all but one patient showed no recurrence. Based on the pathologic diagnosis, the tumor multifocality rate (88.9%) and HGPIN accompanying rate (61.1%) were considerably high, which was a distinct feature in this study compared to the previous studies [7, 22, 23]. This supports the innate characteristics of multifocality in PCa; thus, the message from this descriptive analysis is that we should essentially rule out the co-existence of peripheral zone cancer, which may harbor more aggressive features, using regular follow-up biopsy and mpMRI.

This study had several limitations. First, this was a single-center study, which may not have reflect the heterogeneous treatment policy for IPCa. However, we believe that this study still has a great advantage in offering more conclusive information using the largest prospective registered database with a long follow-up period. Second, less than half of patients diagnosed with IPCa underwent prostate biopsy or prostate MRI before HoLEP. This is partly due to the rapidly increasing role of mpMRI in PCa diagnosis recently. However, we made our best efforts to exclude PCa by applying the PCa risk calculator preoperatively [24]. As a result, the IPCa incidence rate (5.4% in our cohort) was lower than the pooled incidence of 8% reported in a recent systematic review and meta-analysis [5]. Furthermore, patients with pathologically proven BPH and IPCa did not show a difference in PSA levels preoperatively in our cohort compared to previous studies showing higher PSA levels in IPCa [25], indicating well screened patients preoperatively. Moreover, many patients were referred from urology oncologists after excluding the possibility of PCa. Third, due to the limited information, we could not consider socioeconomic status in diagnosis of IPCa, which may have affected the diagnosis and treatment of IPCa. And fourth, patients who underwent AS did not follow a uniform protocol. Most of the patients underwent multiparemetric prostate MRI-based or both MRI and biopsy based surveillance. However, for a certain group of low risk patients (38.1%), PSA-only surveillance was performed after confirmation of no existence of PCa on post-HoLEP follow-up biopsy (S3 Table).

This study is meaningful in that it comprehensively demonstrated the long-term observed natural course of IPCa, which was further analyzed using each subsequent treatment method in the real-world setting. Overall, we revealed that, compared to the previous study, a higher number of patients with IPCa had an intermediate risk (approximately 21%). Patients who underwent AS through the long follow-up period, after approximately 4 years of AS, may be definitive treatment candidates due to ISUP GG increment or core percentage increment, which may also be safely treated (S1 Fig).

## 5. Conclusions

We comprehensively demonstrated the long-term observed natural course of IPCa, which was further analyzed using each subsequent treatment method in the real-world setting. The incidence of IPCa after HoLEP was 5.4%, and among these, approximately 20% proceeded with immediate definitive therapy and an additional 6% ultimately received definitive therapy within the median of 4 years of AS but showed excellent oncological outcomes.

## Supporting information

S1 Dataset (XLSX)

S1 Fig Kaplan-Meier curve of time to the active treatment for incidental prostate cancer after HoLEP surgery. (TIF)

S1 Table Comparison between T1a and T1b incidental prostate cancer. (DOCX)

S2 Table Characteristics and oncological outcome of the active treatment group. (DOCX)

S3 Table Active surveillance protocol in this study. (DOCX)
