# Supplementary figures and images for: Natural history of incidentally diagnosed prostate cancer after holmium laser enucleation of the prostate
Source: PLoS One. 2023 Feb 2;18(2):e0278931. doi: 10.1371/journal.pone.0278931 (PMC9894415; doi:10.1371/journal.pone.0278931)

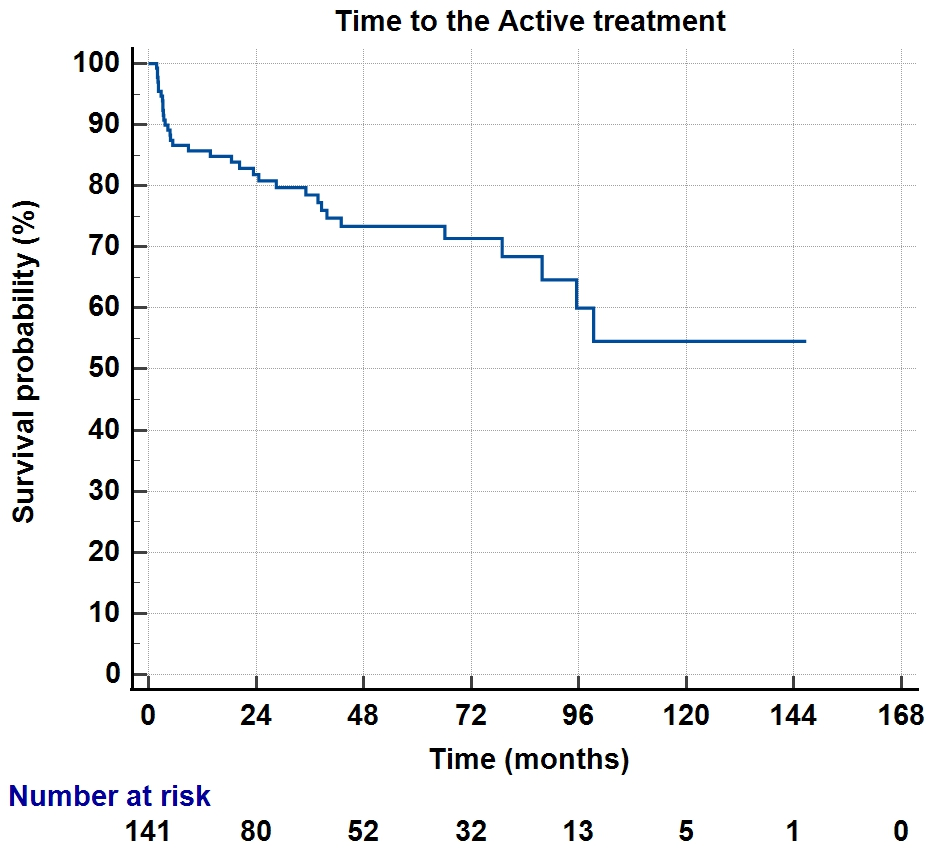

Supplement: S1 Fig — (TIF) [file pone.0278931.s002.tif]
