# Supplementary material for: Natural history of incidentally diagnosed prostate cancer after holmium laser enucleation of the prostate
Source: PLoS One. 2023 Feb 2;18(2):e0278931. doi: 10.1371/journal.pone.0278931 (PMC9894415; doi:10.1371/journal.pone.0278931)
Supplement: S1 Table — (DOCX) [file pone.0278931.s003.docx]

**S1 Table. Comparison between T1a and T1b incidental prostate cancer**

| Number of patients (n) | T1a  (n=125) | T1b  (n=14) | p-value |
| --- | --- | --- | --- |
| Age (years) | 71.8 [68.0, 78.8] | 73.9 [69.9, 78.0] | 0.294 |
| PSA (ng/ml) | 3.2 [1.8, 5.6] | 3.6 [2.6, 6.9] | 0.352 |
| PSA density (ng/ml^2^) | 0.05 [0.03, 0.08] | 0.07 [0.05, 0.16] | 0.054 |
| Total prostate volume | 59.6 [46.5, 77.2] | 51.3 [36.4, 60.4] | 0.069 |
| ISUP Grade Group (n, %) |  |  | 0.389 |
| Grade Group 1 | 107 (85.6) | 11 (78.6) |  |
| Grade Group 2 | 16 (12.8) | 2 (14.3) |  |
| Grade Group ≥3 | 2 (1.6) | 1 (7.1) |  |
| Treatment type (n, %) |  |  | 0.003 |
| Active Surveillance | 90 (83.3) | 5 (41.7) |  |
| Active treatment | 18 (16.7) | 7 (58.3) |  |

†Data are presented as median (interquartile range)

PSA, prostate-specific antigen
