# Supplementary material for: Natural history of incidentally diagnosed prostate cancer after holmium laser enucleation of the prostate
Source: PLoS One. 2023 Feb 2;18(2):e0278931. doi: 10.1371/journal.pone.0278931 (PMC9894415; doi:10.1371/journal.pone.0278931)
Supplement: S2 Table — (DOCX) [file pone.0278931.s004.docx]

**S2 Table. Characteristics and oncological outcome of the active treatment group**

| Total (n) | 25 |
| --- | --- |
| Treatment type (n, %) |  |
| Hormone therapy | 3 (12.0) |
| Radiation therapy | 5 (25.0) |
| Radical prostatectomy | 18 (72.0) |
| Biochemical recurrence (n, %) | 1 |
| Median follow-up period after treatment (n, %) | 88.3 [55.3, 114.4] |
|  |  |
| Pathologic findings (n, %) |  |
| Multifocality | 16 (88.9) |
| Capsular invasion | 2 (11.1) |
| Angiolymphatic invasion | 0 (0.0) |
| Perineural invasion | 5 (27.8) |
| Coexistence of HGPIN | 11 (61.1) |
| ISUP Grade Group (n, %) |  |
| Upgrading (GG1GG2) | 5 (27.8) |
| Identical | 11 (61.1) |
| Downgrading | 2 (11.1) |
| Pathologic T stage (n, %) |  |
| T0 | 1 (5.6) |
| T2 | 17 (94.4) |

†Data are presented as median (interquartile range)

PSA, prostate-specific antigen; PIN, prostate intraepithelial neoplasia; ASAP, atypical small acinar proliferation
