# Supplementary material for: Natural history of incidentally diagnosed prostate cancer after holmium laser enucleation of the prostate
Source: PLoS One. 2023 Feb 2;18(2):e0278931. doi: 10.1371/journal.pone.0278931 (PMC9894415; doi:10.1371/journal.pone.0278931)
Supplement: S3 Table — (DOCX) [file pone.0278931.s005.docx]

**S3 Table. Active Surveillance protocol in this study**

|  | Regular biopsy  every 1-2 years | Regular multiparametric prostate MRI every 1-2years | | PSA follow up after immediate post-op MRI | Number of patients  (n, %) |
| --- | --- | --- | --- | --- | --- |
| Protocol 1 | O | | O | O | 26 (26.8%) |
| Protocol 2 | X | | O | O | 34 (35.1%) |
| Protocol 3 | X | | X | O | 37 (38.1%) |
| Total |  | |  |  | 97 (100%) |

MRI, magnetic resonance imaging; PSA, prostate-specific antigen
